# Toxicology study of a tissue anchoring paclitaxel prodrug

**DOI:** 10.1186/s40360-024-00819-6

**Published:** 2024-12-05

**Authors:** Rukesh Chinthapatla, Jazz Q. Stephens, Isabel B. Neumann-Rivera, Nichol M. Henderson, Minhua Nie, Hannah R. Haynes, Joshua G. Pierce, Danielle M. Meritet, Yevgeny Brudno, Annie Oh

**Affiliations:** 1 Joint Department of Biomedical Engineering, University of North Carolina at Chapel Hill, North Carolina State University, Raleigh, NC USA; 2 Comparative Medicine Institute, North Carolina State University, Raleigh, NC USA; 3 Department of Population Health and Pathobiology, College of Veterinary Medicine, NC State University, Raleigh, NC USA; 4 Department of Chemical and Biomolecular Engineering, North Carolina State University, Raleigh, NC USA; 5 Department of Clinical Sciences, College of Veterinary Medicine, North Carolina State University, North Carolina, Raleigh, USA; 6 Department of Chemistry, North Carolina State University, Raleigh, NC USA; 7 Department of Molecular and Structural Biochemistry, North Carolina State University, Raleigh, NC USA; 8 Lineberger Comprehensive Cancer Center, University of North Carolina at Chapel Hill, Chapel Hill, NC USA; 9 Division of Pharmacoengineering and Molecular Pharmaceutics, Eshelman School of Pharmacy, University of North Carolina at Chapel Hill, Chapel Hill, NC USA; 10 Present Address: 1001 William Moore Drive, Biomedical Partnership Building, Raleigh, NC 27607 USA

**Keywords:** Tissue-Reactive Anchoring Pharmaceuticals (TRAPs), Paclitaxel, Drug delivery, Cremophor, Local chemotherapy

## Abstract

**Background:**

Local drug presentation made possible by drug-eluting depots provides benefits for a vast array of diseases, including cancer, microbial infection, and wound healing. Drug-eluting depots provide sustained drug release of therapeutics directly at disease sites with tunable kinetics, remove the need for drugs to access disease sites from circulation, and reduce the side effects associated with systemic therapy. Recently, we introduced an entirely novel approach to local drug presentation named Tissue-Reactive Anchoring Pharmaceuticals (TRAPs). TRAPs enables local drug presentation without any material carriers, capitalizing on innate tissue structures to anchor drugs at the site of administration.

**Methods:**

In this report, we comprehensively evaluate the local and systemic toxicological profile of a paclitaxel version of TRAPs in mice by clinical observations, body weight monitoring, histopathological evaluations of injection sites and major organs, as well as blood and urine analyses.

**Results:**

We find that intradermal administration of TRAP-paclitaxel does not induce substantial toxic effects. Localized inflammatory responses were observed at the injection sites and secondary minimal, non-specific inflammation was observed in the liver. All other organs displayed unremarkable histological findings.

**Conclusions:**

These findings support the potential of TRAP-paclitaxel as a promising candidate for localized cancer treatment, offering high-concentration drug delivery while mitigating scarring and adverse side effects.

**Supplementary Information:**

The online version contains supplementary material available at 10.1186/s40360-024-00819-6.

## Introduction

Cancer stands as a prominent global contributor to mortality, with an estimated lifetime risk of developing the disease at 40%, and a 20% chance of succumbing to it [1, 2]. While treatments vary based on the cancer type, stage, and patient health, one common challenge persists: the debilitating side effects of chemotherapy drugs [3].

Local drug delivery from drug-eluting depots provides an important healthcare option for cancer in locally-advanced and unresectable areas [4–7] as well as in the resection cavity to prevent recurrence. Drug-eluting depots offer the advantages of high drug doses at disease sites, a sustained drug presence that prevents peaks and troughs in drug presentation, and a continuous drug presence, which improves disease outcomes [8, 9] and patient compliance [10]. Perhaps most importantly, local drug presentation minimizes systemic side effects often seen with systemic drug dosing [11].

Conventionally, local drug delivery devices are placed peritumorally. These include drug-embedded hydrogels and microbeads [12–15], drug-eluting patches [7, 16–18], and infusion devices [19–21]. Unfortunately, these approaches cannot deliver drugs deep into tumors because the tumors’ high stiffness prevents intratumoral injection of viscous materials [22, 23]. This is exemplified by the fact that almost all drug-eluting depots published for pancreatic tumors are designed for peritumoral, rather than intratumoral, application. Thus, despite much clinical and translational research effort, the stiff extracellular matrix (ECM)-rich microenvironment of pancreatic tumors remains a major challenge not only to systemic, but also to local anti-tumor drug presentation.

**Fig. 1:**
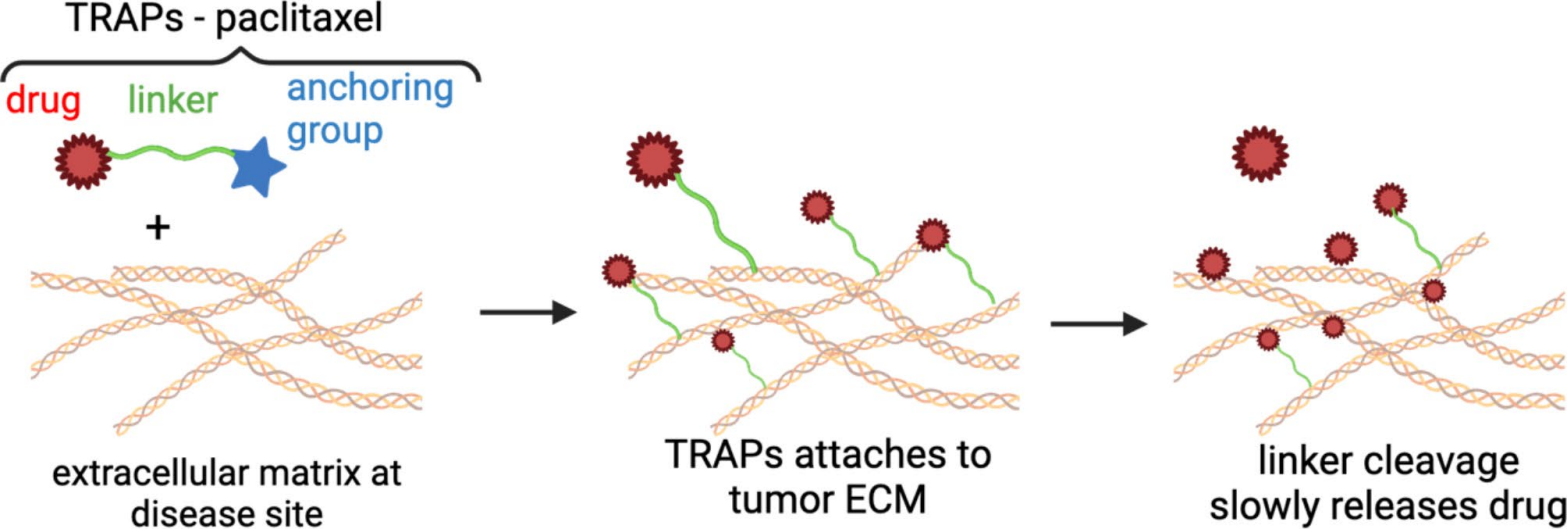
Structures and schematic of TRAP-paclitaxel. Overview of TRAPs technology. TRAP drugs are introduced into disease tissues and consist of a drug connected through a hydrolysable linker to an anchoring motif, which attaches the drug and linker to tissue extracellular matrix. Over time, the ester bonded linking paclitaxel to the succinic acid hydrolyzes, releasing active drugs to the tissue

Our group has recently pioneered a simple, materials-free sustained release technology [24], known as Tissue-Reactive Anchoring Pharmaceuticals (TRAPs, Fig. 1). Rather than using materials to embed a drug, TRAPs takes advantage of tissue structures and anchors drugs to the ECM directly at the site of administration. Using fluorophores, we show these small, reactive molecules penetrate deep into injected tissues, enabling this solution to better infiltrate and spread throughout the whole volume of a target tissue [24, 25]. The drug is anchored to tissue through a robust and tunable chemical linker that slowly dissolves, providing sustained release to the tissue. TRAPs provide a flexible drug delivery platform amenable to many drug classes for the treatment of localized diseases, including cancer, infection, and inflammation.

In prior work, we demonstrated the efficacy of TRAPs-formulated paclitaxel (TRAP-paclitaxel) as a promising therapeutic. Upon local injection, TRAP-paclitaxel diffuses through the tumor and attaches directly to tumor ECM. We showed that TRAP-paclitaxel had enhanced solubility, was easier to administer, provided for controlled release of paclitaxel, and outperformed free paclitaxel in syngeneic mouse models of pancreatic cancer [24, 26–28]. Importantly, continuous presentation of the paclitaxel made possible by the TRAPs system provided superior antitumor efficacy without off-target toxicity.

In this report, we evaluated the local and systemic toxicity of TRAP-paclitaxel. We report improved formulations of TRAP-paclitaxel that minimize local scarring. Detailed toxicology of this formulation found no evidence of systemic and long-term effects of the TRAP-paclitaxel. Taken together, this work establishes injectable TRAP-paclitaxel for further clinical development.

## Methods

### Synthesis of TRAP-paclitaxel

Paclitaxel was purchased from Medkoo Biosciences, Inc. (cat#, 100690). TRAP-paclitaxel was synthesized in a two-step process [24]. Paclitaxel succinic acid was synthesized as previously described [29]. Paclitaxel (Medkoo, cat# 100690) (1 equivalent) was dissolved in dichloromethane (DCM) (Acros Organics, cat# 348465000) under inert conditions and reacted with succinic anhydride (TCI, cat# TCS0107) (2 equivalents) in the presence of (4-Dimethylamino) pyridine (DMAP) (Aldrich, cat# 107700) (1 equivalent). This reaction was allowed to stir overnight at room temperature. The resulting mixture was purified using silica gel chromatography, employing a gradient elution of 10% methanol (MeOH) in DCM. Next, the purified paclitaxel succinic acid (1 equivalent) was subjected to reaction with 1-Ethyl-3-(3-dimethylaminopropyl) carbodiimide (EDC) (Oakwood chemicals, cat# 024810) (1 equivalent) and N-Hydroxysulfosuccinimide (sulfo-NHS) (Combi-Blocks, cat# 82436–78 − 0) (1 equivalent) dissolved in N, N-Dimethylformamide (DMF) (Acros Organics, cat# 348435000) under inert conditions. This reaction was allowed to proceed overnight at room temperature and was followed by purification through ether precipitation (Fisher, cat# AAL14030AU). The final purified paclitaxel-sulfo-NHS conjugate was collected and further characterized using liquid chromatography-mass spectrometry (LC-MS) and nuclear magnetic resonance (NMR) spectroscopy (Figs. S1, S2). The characterization data shows TRAP-paclitaxel product was > 95% pure.

### Drug formulation

N-Methyl-2-pyrrolidone (NMP) was purchased from (Alfa Aesar, cat#AA44063-K2). Physiological saline was purchased from VWR (cat#, 470302-026). For NMP formulation, paclitaxel (8 mg/mL) or TRAP-paclitaxel (20 mg/mL) was readily dissolved in NMP before diluting with equal parts saline. For Cremophor EL (CrEL) formulation (10% Cremophor EL, 10% EtOH, 80% D5W), paclitaxel (8 mg/mL) or TRAP-paclitaxel (20 mg/mL), was combined with ethanol and sonicated to give a saturated solution with a white paste-like appearance. Separately, a combination of sulfo-NHS (3.45 mg/mL) and succinic anhydride (1.77 mg/mL) was combined in ethanol resulting in an equimolar concentration to TRAP-paclitaxel. Cremophor EL was purchased from Millipore Sigma (cat#, 238470). Dextrose 5% in water was prepared in house using dextrose from Fisher Scientific (cat#, D16-500). To this solution, an equal volume Cremophor EL was added, and the mixture dissolved by gentle pipetting. The solution was finally diluted 5-fold in dextrose 5% in water prior to administration.

### Animal studies

All animal work was done in compliance with Institutional Animal Care and Use Committee (IACUC; 22-233-O; Approved 06.17.2022) policies including the National Institutes of Health’s Guide for Care and Use of Laboratory Animals, National Research Council’s Guide for the Care and Use of Laboratory Animals, and Animal Research: Reporting of In Vivo Experiments (ARRIVE) guidelines. 12-week old mice (CD-1, non-pigmented) were purchased from Charles River Laboratories (Morrisville, NC) and housed in ventilated cages with access to food and water. All formulations were prepared immediately before administration. Mice were anesthetized with isoflurane (4–5% induction, 1–2% maintenance), and the skin was shaved with a hair trimmer and disinfected using an alcohol wipe before injection. Intradermal injections were placed caudal to the scapula with an insulin syringe and a 27-gauge needle. A single dose was administered to each mouse depending on the assigned group.

For NMP formulation, two treatment groups were assessed with each group containing randomly assigned mice: (1) saline (control, 50 µL; female *n* = 3 and male *n* = 3) and (2) NMP (vehicle, 50 µL; female *n* = 6 and male *n* = 5). For CrEL formulation (10% Cremophor EL, 10% EtOH, 80% D5W), five treatment groups were assessed with each group containing randomly assigned female (*n* = 4) and male (*n* = 4) mice: (1) saline (control; 10 µL), (2) CrEL (vehicle; 10 µL), (3) paclitaxel with CrEL (20 mg/kg, 8 mg/mL), (4) TRAP (3.45 mg/mL sulfo-NHS and 1.77 mg/mL succinic anhydride; 10 µL), and (5) TRAP-paclitaxel with CrEL (50 mg/kg, 20 mg/mL).

### Monitoring of injection site and body weight

Mice were examined and photographed daily using a Nikon digital SLR camera for 4 weeks. At the end of weeks three and four, the injection site was shaved and disinfected with alcohol wipes for a better view of the skin. Body weights were recorded daily.

### Collection of blood, urine, and tissues

Before the start of toxicology study, blood (0.1 mL) and urine (0.05–0.5 mL) were collected from retro-orbital bleeding and sacral vertebral stimulation, respectively. At the end of the toxicology study (4-weeks after treatment), urine was collected similarly. Following, the mice were euthanized and blood was collected via cardiac puncture (0.5–1.5 mL) using a 25-gauge needle following euthanasia. Blood and urine samples were pooled per group and submitted for further analytical processing (NC State Clinical Pathology Lab).

The injection site was shaved and photographed. Subsequently, the injection site and surrounding skin was excised, placed flat in cassettes, and immediately fixed in 10% neutral buffered formalin. Organs were collected during necropsy and immediately fixed in 10% neutral buffered formalin. All tissue samples from three representative samples of each gender were submitted for further histological processing (Histology Laboratory at NC State College of Veterinary Medicine). Hematoxylin and eosin (H&E) stained sections were imaged at 40x magnification (Olympus VS200 Slide Scanner) and sent to two blinded reviewers, a board-certified pathologist and pathology resident, for evaluation.

### Statistical analysis

All data was presented and analyzed for statistical significance using Prism software (version 9.0.1). To evaluate the statistical significance for body weight change, ordinary one-way analysis of variance (ANOVA) was used.

## Results

### Improving vehicle formulation for injection of TRAP-paclitaxel

Paclitaxel has very low solubility in aqueous solutions (< 2 µg/mL), posing a challenge for drug administration [27, 30]. Our previous study [24] used a 1:1 combination of saline and NMP to solubilize the paclitaxel and improve TRAP-paclitaxel solubility. Although NMP has been used clinically [31], it has been reported to be cytotoxic [32], and indeed we observed a local necrotic response in the NMP-alone group [24].

For clinical use of TRAP-paclitaxel and especially for use in the tumor resection bed, we believe that immediate local toxicity as seen with NMP might be undesirable. To quantify the extent of local NMP toxicity, we tested the saline/NMP mix by assessing tissue reaction after intradermal injection in outbred, immunocompetent CD1 mice. Although NMP in small doses is considered non-irritating, is used as a skin permeability enhancer [33], and the mice did not show any weight differences (Fig. S3), we concluded that clinically relevant doses would cause significant ulcerations and scarring to the skin that is intolerable, especially in sensitive areas (Fig. S4).

We sought to minimize the adverse local effects of NMP injection by reformulating the vehicle. Aqueous solutions using dextrose, ethanol, and Cremophor EL (CrEL) are already approved for paclitaxel formulation and used in intravenous treatment of Kaposi’s sarcoma, ovarian, breast, and lung cancers, as well as off-label use for several additional cancers [34]. CrEL and ethanol both enhance solubility and stabilize the drug in solution, preventing precipitation and aggregation of paclitaxel particles.

We evaluated CrEL formulations of the TRAPs system for intradermal administration. TRAP-paclitaxel, free paclitaxel, and the individual TRAP components (succinic anhydride and sulfo-NHS, Fig. 1) were formulated using equal parts CrEL and ethanol diluted 5-fold (10% CrEL) in a 5% dextrose water solution. Similarly to our original studies with NMP, we achieved solubilities of 8 mg/mL for paclitaxel and 20 mg/mL for TRAP-paclitaxel. The TRAPs solutions were stable, with no precipitation observed. The paclitaxel solution was stable for over 30 min but incubating for periods longer than that led to precipitation marked by a white haze in the solution.

### Intradermal injections led to broadly normal clinical observations and body weight

**Fig. 2:**
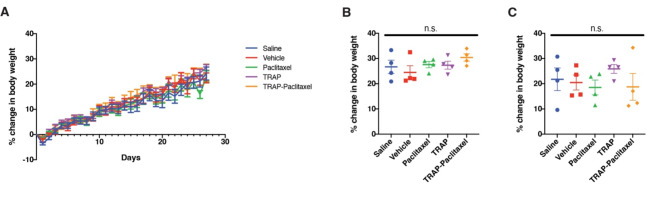
Animal weights show no difference between control and experimental groups with Cremophor EL (CrEL) formulation. **A** Daily percent change in body weight of mice over duration of toxicology study. **B** Male and **C** female comparison of percent change in body weight between treatment groups at end of study. Statistical analysis performed by one-way ANOVA

With the new formulation in hand, we tested the local and systemic effects of intradermal administration of the vehicle, free paclitaxel, TRAP paclitaxel, and the individual components making up the TRAP system (Fig. S5). Paclitaxel was dosed at its solubility limit (9.37 mM, 8 mg/mL), while the other components were more soluble and could be dosed at a higher concentration (17.68 mM, 20 mg/mL). No concerns regarding clinical observations were noted in any of the treatment groups. All mice survived and were bright, alert, responsive, and in good condition. No indication of pain or distress was noticed, and behavior was normal. All mice gained weight as expected throughout the one-month study. Natural variation in percent change of body weight of mice in all groups was observed and ranged from 10 to 34% increase compared to initial body weight (Fig. 2A). No statistically significant differences between treatment groups (males and females alone or combined) were observed by one-way ANOVA at the end of the 4-week study (Fig. 2B, C).

**Fig. 3:**
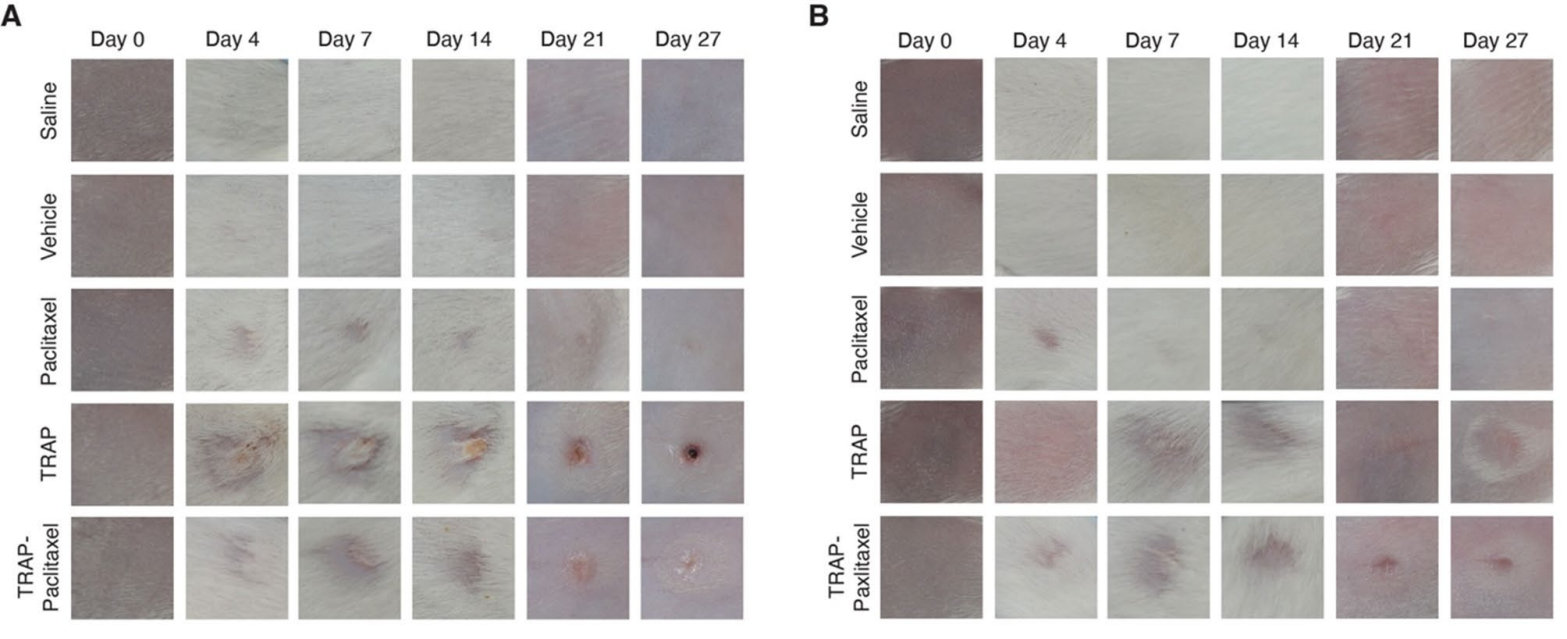
Minimal site reactivity upon intradermal injections of all treatment groups with Cremophor EL (CrEL) formulation. **A** Male and **B** female groups of saline, vehicle (10% CrEL, 10% EtOH, 80% D5W), paclitaxel (8 mg/mL), TRAP components (3.45 mg/mL sulfo-NHS and 1.77 mg/mL succinic anhydride), and TRAP-paclitaxel (20 mg/mL) were intradermally injected in a volume of 10 µL. Injection sites of mice receiving saline, vehicle, or paclitaxel were unremarkable. The formation of scabs and alopecia were observed in the TRAP and TRAP-PTX groups. Representative images from one mouse per group are shown. Mice were shaved on day 0 and again on days 21 and 27. Images are 1 cm x 1 cm

Direct observation of the injection site provided crucial insights into the local tissue response following exposure to the therapy. Alopecia was observed in both female and male mice receiving TRAP and TRAP-paclitaxel, and to a lesser degree in the paclitaxel group after intradermal administration (Fig. 3). The hair loss is considered an expected side effect of taxane chemotherapy [35]. Mice in the TRAP and TRAP-paclitaxel groups also formed an eschar (scab) at the injection site within a week of receiving treatment, which was generally more pronounced in the TRAP-paclitaxel group than the TRAP-only group. The affected area was roughly 9 mm^2^ in size. By the end of two weeks, debridement of the scabs revealed small ulcers that were mostly closed and a pink-red color due to new formation of skin during local wound healing. Minor scarring was observed at three weeks in the TRAP and TRAP-paclitaxel groups. Surprisingly, hair growth was seen directly on the scabs, but not the surrounding area, and faster hair regrowth at the injection site was noted in these mice. Some mice in all groups developed a rash due to irritation from repeated shaving distant from the injection site.

### Histological evaluation of the injection site revealed minimal to moderate inflammation

**Fig. 4:**
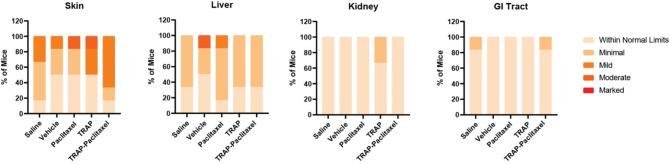
Summarized histopathology grading show increased inflammation at injection site of depot. Stacked bar charts show composition of inflammation score of experimental and control groups by organs. The skin and liver showed most significant and consistent findings of inflammation. Two blinded histopathologists individually graded all tissues of euthanized mice for inflammation and necrosis

Evaluation of the injection site is vital for assessing local safety by identifying potential adverse reactions and ensuring the overall safety of the locally eluting depots for clinical use. Histopathological image analysis by two blinded, independent pathologists revealed minimal to moderate inflammation (grades 1–2) in 63.3% of the mice (Fig. 4, S6). Panniculitis was seen in 17 of 30 mice spread evenly across all experimental groups with inflammatory cells varying but typically ranged from scattered macrophages and granulocytes to predominantly mononuclear cells. Granulomatous inflammation around free keratin (foreign-body type response), a type of chronic inflammation, was seen in two male mice that received TRAP-paclitaxel one of which displayed evidence of scar tissue. These responses are considered secondary to the injection. As expected, mice in the TRAP-paclitaxel group displayed more inflammation compared to saline and vehicle controls. The frequency of inflammation was also higher than the free paclitaxel group, likely due to the higher concentration of paclitaxel administered (17.68 vs. 9.37 mM). Half the mice in the TRAP group displayed mild to moderate inflammation suggesting the TRAP components (sulfo-NHS and succinic anhydride) may also contribute to inflammation seen in the TRAP-paclitaxel groups.

### Histological evaluation of major organs following TRAPs administration was unremarkable

Histological evaluation of organs is crucial for assessing off-target toxicity on specific tissues and supports guidance for the development and clinical use of the drug. Since the liver, kidney, and gastrointestinal (GI) tract are commonly affected by paclitaxel associated toxicity we examined these organs for adverse effects [36, 37]. Some inflammation was observed in the livers of mice from all groups, but TRAP and TRAP-paclitaxel groups scored within normal limits to minimal levels of inflammation (Fig. 4, S7). One mouse in the paclitaxel group scored mild inflammation while one mouse in the vehicle group showed moderate inflammation and all other mice exhibited within normal limits or minimal levels of inflammation. The clinical formulation of paclitaxel is associated with hepatoxicity [38]. Although Cremophor is not fully inert and exerts a range of effects, specific toxicity in the liver has not previously observed [39]. Rare small clusters of hepatocellular death with satellitosis were found in the saline, vehicle, paclitaxel, and TRAP component experimental groups. This single cell death likely represents apoptosis from homeostatic cell turnover. Additionally, rare spontaneous hepatocellular necrosis within regions of inflammation was found in one saline-treated and one vehicle treated mouse. The saline-treated mouse with hepatocellular necrosis showed neutrophilic inflammation in the regions of focal necrosis but is considered secondary to the necrosis. Vacuolation was also observed in these necrotic cells. The necrosis is thought to be caused by potential hypoxia during restraint and handling with isoflurane and unrelated to the treatments. Focal mononuclear aggregates were found in 13 of the 30 mice from all groups and are considered background lesions. Overall, the hepatic inflammation observed may reflect a minimal, non-specific systemic pro-inflammatory response.

Evaluation of the kidney and gastrointestinal tract (stomach, small and large intestine) was within normal limits to demonstrated minimal inflammation (Fig. 4). No significant findings were found in either the gastrointestinal tract or kidneys.

### Bloodwork and urine analysis values show no gross changes

Bloodwork, including complete blood count (CBC) with cell differentiation, clinical biochemistry parameters, and coagulation tests are important in assessing the impact of the drug on the hematopoietic system, organ function, and overall safety. To assess these values and ensure sufficient volume, blood was pooled from both all mice in each treatment group.

CBC and cell differential values mostly showed no gross changes in any of the parameters at the study start and end. Summary of CBC and cell differentiation parameters are presented in Table S1. Red cell distribution width, reticulocyte % and absolute count decreased at the end of the study period and plasma protein was slightly increased in all treatment groups, indicating no treatment-specific effects.

Clinical biochemistry values mostly showed no significant differences in any of the parameters at the study start and end (Table S2). Bicarbonate levels, glucose levels, and hemolysis were increased while creatine kinase was decreased in all groups. Interestingly, significantly increased lipase and amylase levels were found in the vehicle group, potentially indicating a pancreatic issue. However, as this finding was consistently absent in the other treatment groups which used the same vehicle, this may be a technical error.

Urine analysis is a valuable component of toxicology studies as it helps monitor nephrotoxicity, assess renal function, identify kidney toxicity, and evaluate metabolite excretion. We therefore collected urine via sacral vertebral stimulation before administering treatment for baseline values, and immediately before euthanasia at the study end. Urine from all mice were pooled by their respective groups. No gross changes in urine were identified before and after the treatment administration. Urinalysis revealed no signs of toxicity in the TRAP-paclitaxel group (Table S4). The pH, glucose, ketones, bilirubin levels, and urine color and clarity were normal. For unknown reasons the protein dipstick and blood levels, were significantly increased in the saline group. The treatment groups, otherwise, presented normal clinical findings.

## Discussion

In this study, we assessed the toxicological impacts of Tissue-Reactive Anchoring Pharmaceuticals (TRAPs) conjugated to the chemotherapeutic agent paclitaxel. Previous work has highlighted that the small size of TRAPs molecules enables broad tissue diffusion after intratumoral injection in both human and mouse pancreatic tumors, and TRAP-paclitaxel induced higher tumoral apoptosis and sustained better antitumor efficacy than free paclitaxel in mouse pancreatic tumors [24]. TRAPs creates local, drug delivering depots through a novel mechanism—direct conjugation of drugs to the extracellular matrix—and releases drugs in a sustained manner through hydrolytic degradation. Further studies are needed to delineate TRAPs distribution with a variety of tumor types and locations. In this study, we sought to advance TRAPs through preclinical development and position TRAPs potential drug delivery modality for localized cancer treatment by assessing its toxicity and tolerability. The urgent need for potent local treatments of unresectable tumors and for prevention of local recurrence is well-recognized in oncology, and TRAPs offer a promising solution to this challenge.

Our toxicological evaluation provides additional insights into the safety attributes of TRAP-paclitaxel. Initial formulation utilizing NMP as the vehicle resulted in significant local ulceration and scarring of normal skin. Therefore, reformulation with CrEL and ethanol was pursued, which restored local tolerance in intradermal murine models. This improvement is pivotal, suggesting that TRAP-paclitaxel, due to its minimal local adverse reactions, stands as a viable candidate for clinical use.

In order to further the clinical development of this compound, we focused on possible intradermal and systemic toxicity. Observations from clinical assessment, body weight, histological examinations, and biochemical analysis of blood and urine demonstrated no substantial issues linked to TRAP-paclitaxel administration, although this study’s one-month timeline implies that acute toxic alterations remain possible. The overall interpretation, coupled with the general well-being of the study’s mice, suggests at the promising safety profile of TRAP-paclitaxel.

## Conclusion

This study establishes that the intradermal administration of TRAP-paclitaxel is well tolerated in mice, inducing no noteworthy toxic effects. As anticipated, the predominant inflammatory responses were localized to the injection sites, with secondary minimal non-specific inflammation observed in the liver. The examinations of all other organs were largely unremarkable. The 10% CrEL formulation minimizes local scarring and attains improved solubility for both paclitaxel and TRAP-paclitaxel compared to our previous formulation containing NMP. These findings synergize with previous work to advance tissue-reactive pharmaceuticals as a treatment modality for locally advanced unresectable tumors with the promise of maintaining localized high-concentration of drug doses while mitigating scarring and toxic effects [24]. The next phase of this research will pivot towards assessing the efficacy of TRAP-paclitaxel in clinical studies, targeting the treatment of locally advanced unresectable tumors and resection beds and furthering our understanding of its potential role and benefits in oncological therapeutics.

## Electronic supplementary material

Below is the link to the electronic supplementary material.

Supplementary Material 1

## Data Availability

The datasets used and/or analyzed during the current study are available from the corresponding author on reasonable request.

## Abbreviations

ANOVA: Analysis of variance
ARRIVE: Animal Research: Reporting of In Vivo Experiments
CBC: Complete blood count
CrEL: Cremophor EL
DCM: Dichloromethane
DMAP: 4-Dimethylamino pyridine
DMF: N, N-Dimethylformamide
ECM: Extracellular matrix
EDC: 1-Ethyl-3-(3-dimethylaminopropyl) carbodiimide
GI: Gastrointestinal
H&E: Hematoxylin and eosin
IACUC: Institutional Animal Care and Use Committee
LC-MS: Liquid chromatography-mass spectrometry
MeOH: 10% methanol
NMP: N-Methyl-2-pyrrolidone
NMR: Nuclear magnetic resonance
Sulfo-NHS: N-Hydroxysulfosuccinimide
TRAPs: Tissue-Reactive Anchoring Pharmaceuticals
