# Supplementary material for: Toxicology study of a tissue anchoring paclitaxel prodrug
Source: BMC Pharmacol Toxicol. 2024 Dec 5;25:92. doi: 10.1186/s40360-024-00819-6 (PMC11619278; doi:10.1186/s40360-024-00819-6)
Supplement: Supplementary file 1 — Supplementary Material 1 [file 40360_2024_819_MOESM1_ESM.docx]

**Supplementary Information**


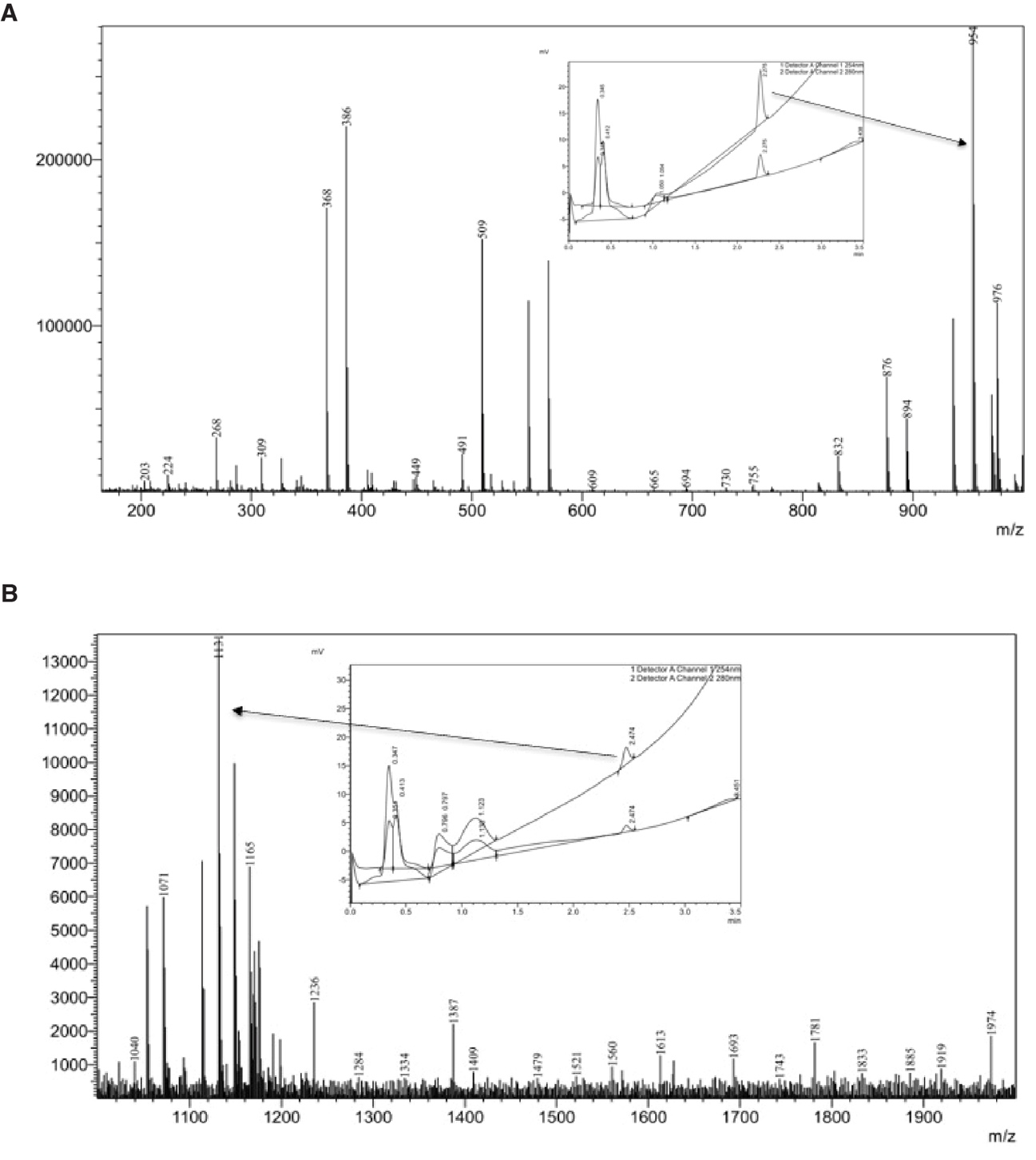


**Figure S1: LC-MS analysis of structures.** (A) purified paclitaxel succinate (B) purified

TRAP paclitaxel.


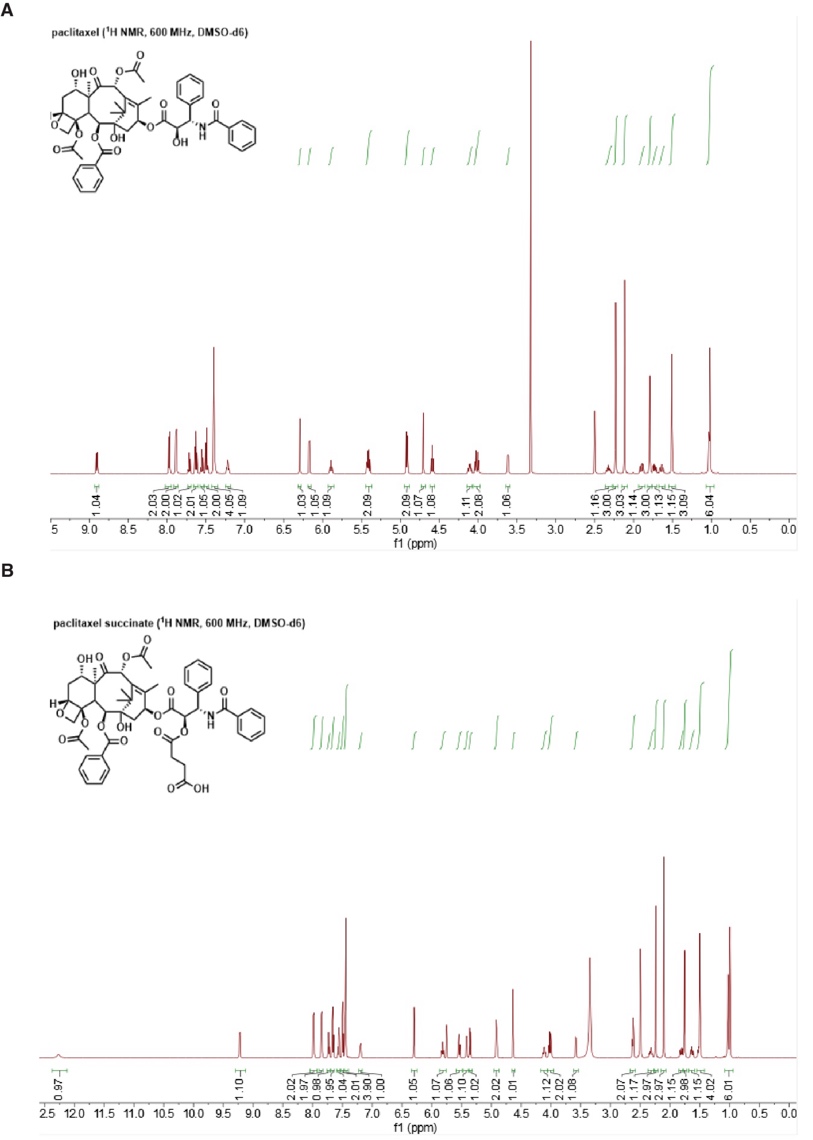

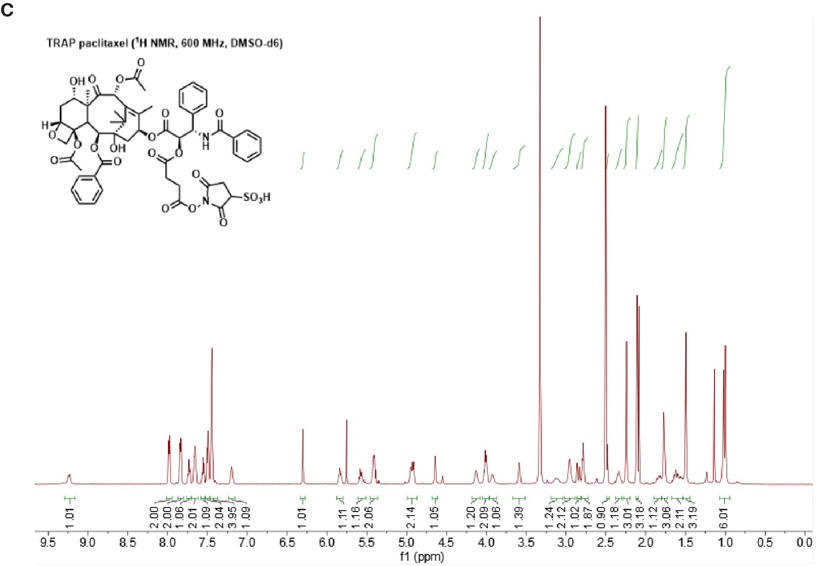


**Figure S2: NMR analysis of structures.** (A) paclitaxel (B) purified paclitaxel succinate (C)

purified TRAP paclitaxel.

**Figure S3. Animal weight monitoring shows no difference between control and experimental group with NMP vehicle.** (A) Daily percent change in body weight of mice throughout the toxicology study. (B) Comparison of percent change in body weight at the end of the study. Statistical analysis was performed by one-way ANOVA.


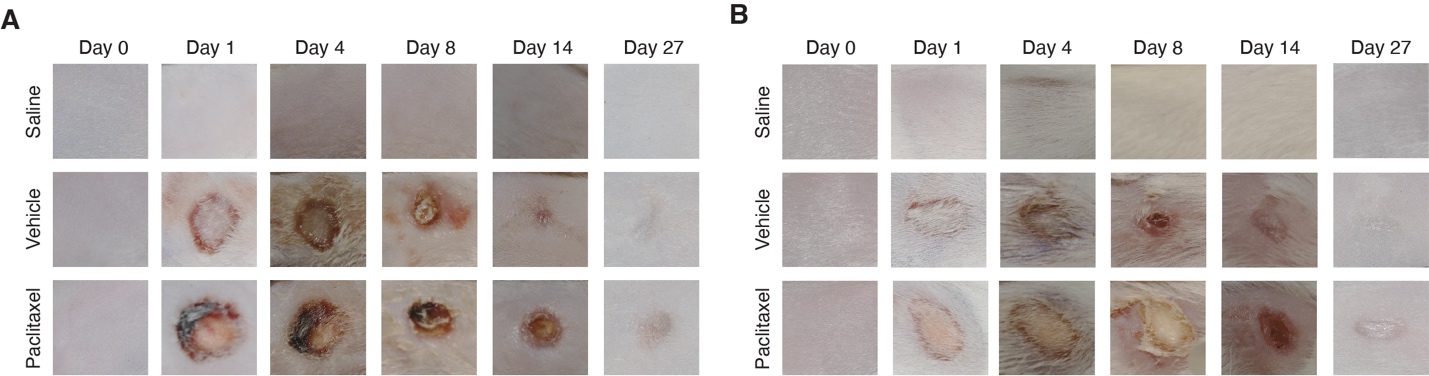


**Figure S4. Macroscopic images after intradermal injection show NMP vehicle causes large ulcerations.** (A) Male and (B) female groups of saline control or vehicle (50% NMP) were intradermally administered at a volume of 50 µL. Injection sites of mice in the saline group are unremarkable. Saline and NMP vehicle groups show ulcerations within 24 hours. All wounds healed within one month from the day of injection. Representative images from one mouse per group are shown. Images are 1 cm x 1 cm.


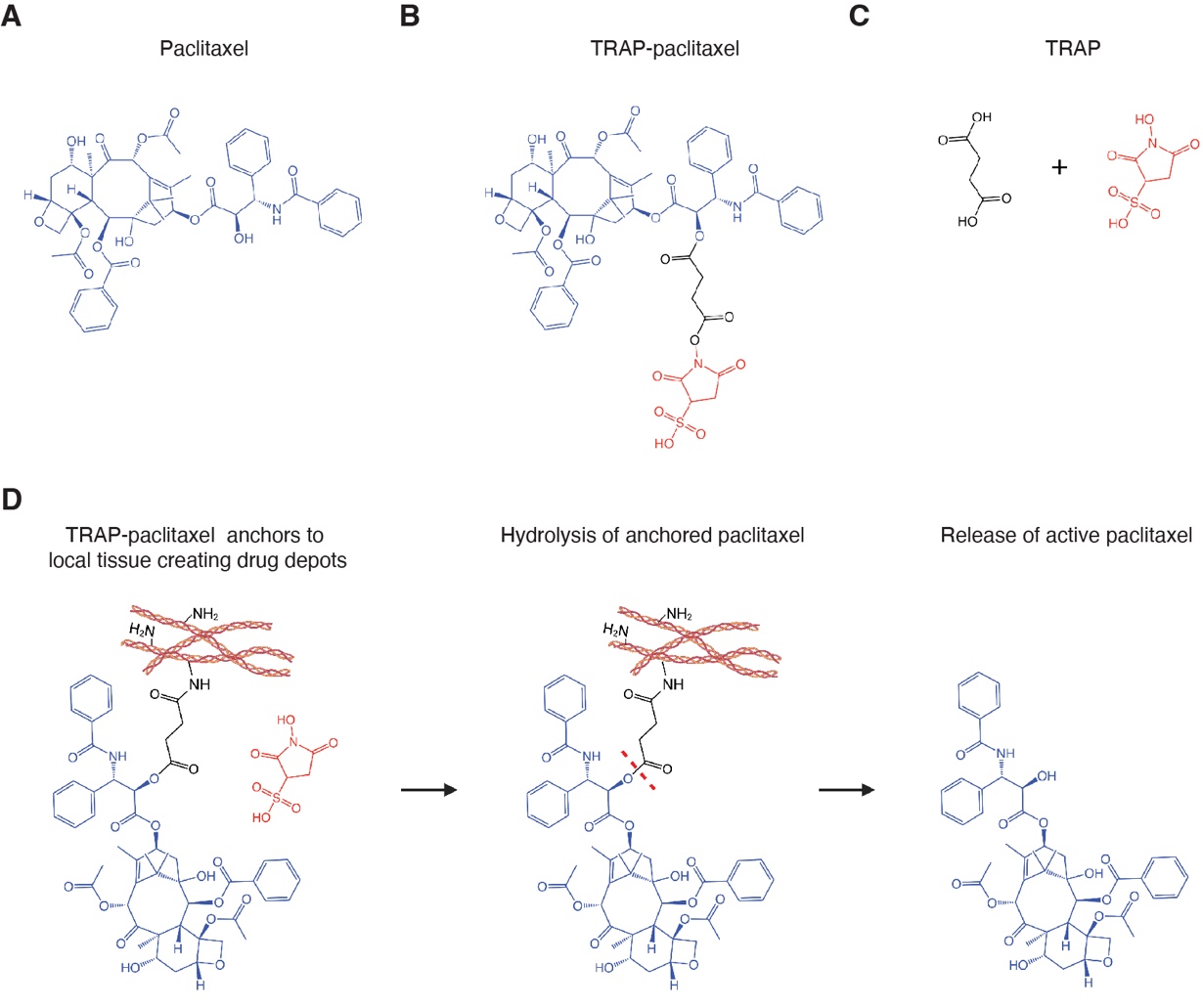


**Figure S5. Structures and schematic of TRAP-paclitaxel.** Structure of (A) paclitaxel, (B) TRAP-paclitaxel, and (C) TRAP components: sulfo-NHS (anchor) and succinic anhydride (linker). (D) Schematic showing fate of TRAP-paclitaxel after intradermal injection. Paclitaxel anchored to extracellular matrix via amine bond and sulfo-NHS as the releasing group, followed by hydrolysis of anchored paclitaxel to release active paclitaxel in the local environment.


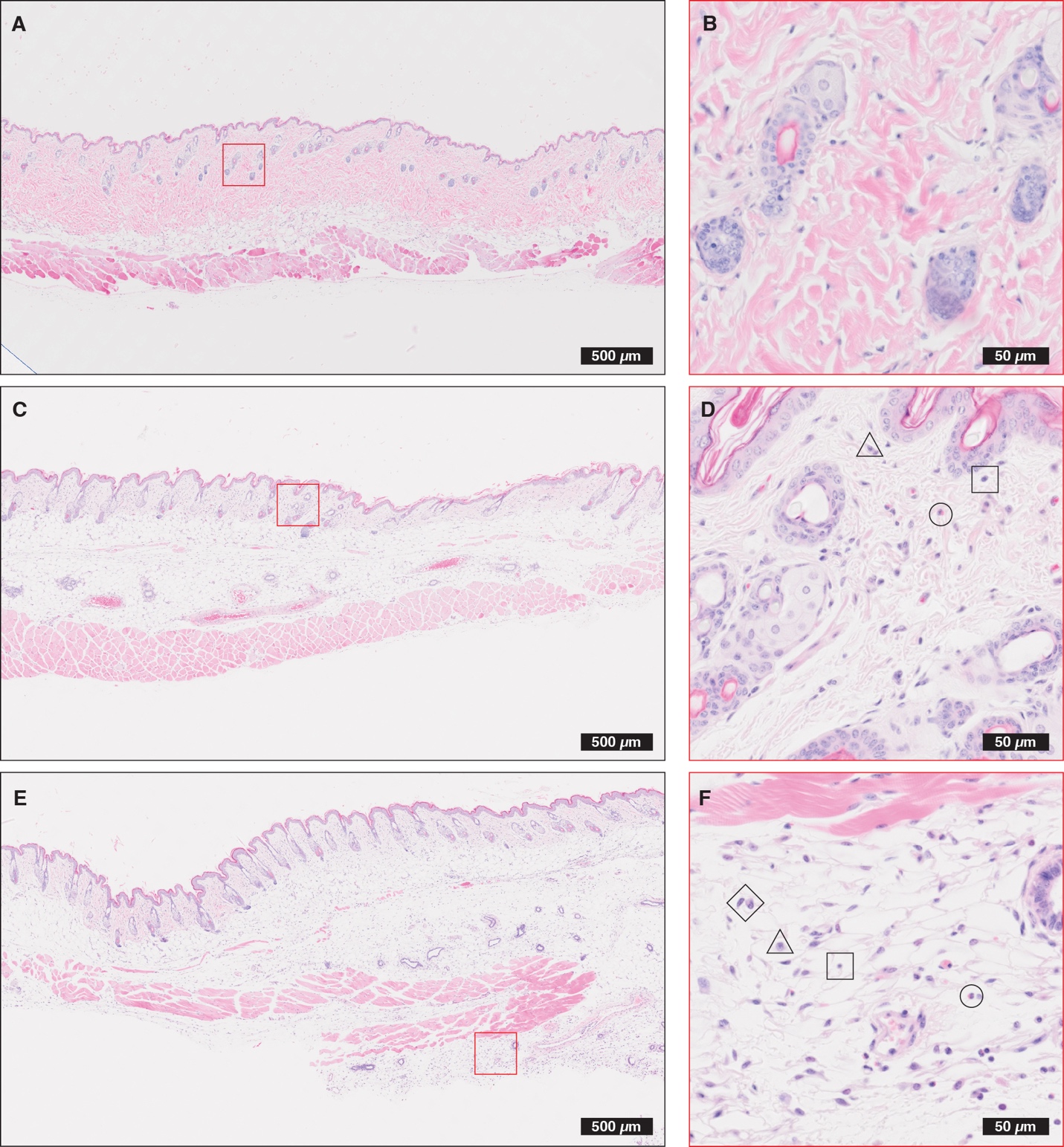


**Figure S6. Representative images of normal, mild, and moderate inflammation at the injection site (skin) of mice.** Male mouse from TRAP group, female mouse from TRAP-paclitaxel group, and female mouse from paclitaxel group show normal (A & B), mild (C & D), and moderate inflammation (E & F), respectively. Legend: plasma cells (diamond), macrophages (triangle), eosinophils (circle), and lymphocyte (square).


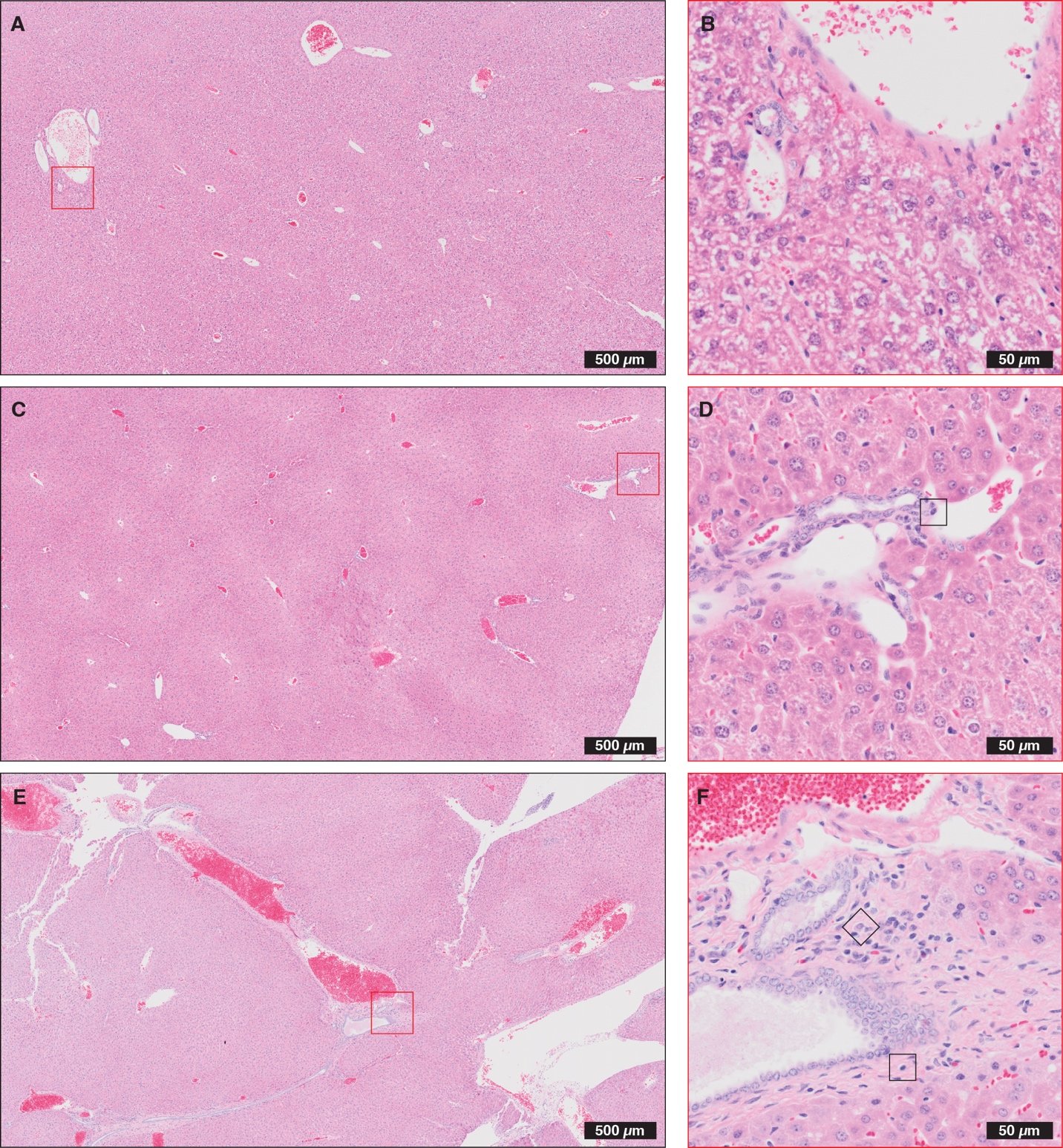


**Figure S7. Representative images of normal, mild, and moderate inflammation in the livers of mice.** Male mouse from TRAP-paclitaxel group, female mouse from TRAP-paclitaxel group, and female mouse from Cremophor EL (CrEL) vehicle group show normal (A & B), mild (C & D), and moderate inflammation (E & F), respectively. Legend: plasma cells (diamond), macrophages (triangle), eosinophils (circle), and lymphocyte (square).

**Supplementary Table 1: Complete blood count (CBC) with cell differentiation**

|  |  | *Saline* | |  | | *Vehicle* | | |  | | *Paclitaxel* | | |  | *TRAP* | |  | *TRAP-Paclitaxel* | | |  | |
| --- | --- | --- | --- | --- | --- | --- | --- | --- | --- | --- | --- | --- | --- | --- | --- | --- | --- | --- | --- | --- | --- | --- |
| TEST | International Units (IU) | Day 0 | Day 28 | |  | | Day 0 | Day 28 | |  | | Day 0 | Day 28 |  | Day 0 | Day 28 | |  | Day 0 | Day 28 | |  |
| *White Blood Cell* | 10^3^/µL | 4.57 | **5.51** | |  | | 5.38 | **5.81** | |  | | 7.03 | **8.05** |  | 6.86 | **4.56** | |  | 6.01 | **9.55** | |  |
| *Red Blood Cell* | 10^6^/µL | 8.53 | **10.8** | |  | | 8.5 | **10.96** | |  | | 8.88 | **11.03** |  | 8.73 | **8.94** | |  | 8.62 | **10.25** | |  |
| *Hemoglobin* | g/dL | 14.8 | **17.5** | |  | | 14.8 | **17.6** | |  | | 14.8 | **18.3** |  | 14.8 | **14.6** | |  | 14.6 | **16.8** | |  |
| *Hematocrit* | % | 49.3 | **58** | |  | | 49.7 | **59.3** | |  | | 50.2 | **60.3** |  | 51.3 | **48.4** | |  | 48.5 | **56.5** | |  |
| *Mean Corpuscular Volume* | fL | 57.9 | **57.3** | |  | | 58.5 | **54.1** | |  | | 56.5 | **54.7** |  | 58.7 | **54.1** | |  | 56.3 | **55.1** | |  |
| *Mean Corpuscular Hemoglobin* | pg | 17.3 | **16.2** | |  | | 17.4 | **16** | |  | | 16.6 | **16.6** |  | 17 | **16.3** | |  | 16.9 | **16.4** | |  |
| *Mean Corpuscular Hemoglobin Concentration* | g/dL | 30 | **30.1** | |  | | 29.8 | **29.6** | |  | | 29.4 | **30.3** |  | 28.9 | **30.2** | |  | 30.1 | **29.8** | |  |
| *Red Cell Distribution Width* | % | 13.8 | **11.2** | |  | | 13.6 | **11.3** | |  | | 14.2 | **11.7** |  | 14.1 | **10.9** | |  | 13.3 | **10.7** | |  |
| *Reticulocyte %* | % | 6.84 | **3.6** | |  | | 6.88 | **2.61** | |  | | 6.21 | **2.81** |  | 9.75 | **3.7** | |  | 6.08 | **2.33** | |  |
| *Reticulocyte #* | 10^6/µL | 0.548 | **0.389** | |  | | 0.584 | **0.286** | |  | | 0.552 | **0.31** |  | 0.852 | **0.33** | |  | 0.524 | **0.239** | |  |
| *Reticulocyte, Absolute* | /UL | 584000 | **389000** | |  | | 548000 | **286000** | |  | | 552000 | **310000** |  | 852000 | **330000** | |  | 524000 | **239000** | |  |
| *Segmented Neutrophils* | 10^3^/UL | 0.548 | **0.937** | |  | | 0.592 | **0.639** | |  | | 0.984 | **1.047** |  | 0.48 | **0.638** | |  | 0.841 | **0.764** | |  |
| *Lymphocytes* | 10^3^/UL | 3.656 | **4.243** | |  | | 4.25 | **4.764** | |  | | 5.694 | **6.44** |  | 5.488 | **3.648** | |  | 4.628 | **8.022** | |  |
| *Monocytes* | 10^3^/UL | 0.229 | **0.11** | |  | | 0.377 | **0.116** | |  | | 0.211 | **0.161** |  | 0.617 | **0.046** | |  | 0.361 | **0.191** | |  |
| *Eosinophils* | 10^3^/UL | 0.137 | **0.11** | |  | | 0.161 | **0.291** | |  | | 0.14 | **0.403** |  | 0.14 | **0.228** | |  | 0.601 | **0.573** | |  |
| *PCV* | % | 48 | **57** | |  | | 48 | **59** | |  | | 48 | **57** |  | 48 | **45** | |  | 47 | **52** | |  |
| *Plasma Protein* | g/dl | 6 | **6.9** | |  | | 5.7 | **7.3** | |  | | 5.7 | **7.1** |  | 5.7 | **6** | |  | 5.6 | **6.5** | |  |

**Supplementary Table 2: Clinical biochemistry parameters**

|  |  | *Saline* | |  | *Vehicle* | |  | *Paclitaxel* | |  | *TRAP* | |  | *TRAP-Paclitaxel* | |
| --- | --- | --- | --- | --- | --- | --- | --- | --- | --- | --- | --- | --- | --- | --- | --- |
| TEST | International Units (IU) | Day 0 | Day 28 |  | Day 0 | Day 28 |  | Day 0 | Day 28 |  | Day 0 | Day 28 |  | Day 0 | Day 28 |
| *Glucose* | mg/dL | 152 | **210** |  | 174 | **245** |  | 174 | **258** |  | 171 | **241** |  | 172 | **267** |
| *Urea Nitrogen* | mg/dL | 29 | **31** |  | 27 | **23** |  | 26 | **27** |  | 28 | **31** |  | 27 | **28** |
| *Creatinine* | mg/dL | <0.2 | **<0.2** |  | <0.2 | **<0.2** |  | <0.2 | **<0.2** |  | <0.2 | **<0.2** |  | <0.2 | **<0.2** |
| *Phosphorus* | mg/dL | 11.2 | **12.4** |  | 10.8 | **12.8** |  | 12 | **12.4** |  | 11.4 | **11.6** |  | 12.4 | **11.6** |
| *Calcium* | mg/dL | 10.8 | **11.5** |  | 10.6 | **11.5** |  | 10.6 | **11.5** |  | 10.6 | **11.1** |  | 11.2 | **11.4** |
| *Magnesium* | mg/dL | 2.6 | **3.4** |  | 2.8 | **2.9** |  | 3.1 | **3.2** |  | 2.7 | **3.1** |  | 2.9 | **3.4** |
| *Total Protein* | g/dL | 4.9 | **5.7** |  | 4.8 | **5.6** |  | 4.9 | **5.5** |  | 4.7 | **5.3** |  | 4.9 | **5** |
| *Albumin* | g/dL | 3.6 | **4.1** |  | 3.4 | **4.2** |  | 3.6 | **4.1** |  | 3.5 | **3.9** |  | 3.6 | **3.8** |
| *Globulin* | g/dL | 1.3 | **1.6** |  | 1.4 | **1.4** |  | 1.3 | **1.4** |  | 1.2 | **1.4** |  | 1.3 | **1.2** |
| *Albumin/Globulin Ratio* |  | 2.77 | **2.56** |  | 2.43 | **3** |  | 2.77 | **2.93** |  | 2.92 | **2.79** |  | 2.77 | **3.17** |
| *Cholesterol* | mg/dL | 143 | **165** |  | 135 | **156** |  | 135 | **158** |  | 129 | **141** |  | 130 | **153** |
| *Total Bilirubin* | mg/dL | <0.2 | **<0.2** |  | <0.2 | **<0.2** |  | <0.2 | **<0.2** |  | <0.2 | **<0.2** |  | <0.2 | **<0.2** |
| *Alkaline Phosphatase* | IU/L | 200 | **122** |  | 188 | **117** |  | 219 | **151** |  | 187 | **132** |  | 239 | **182** |
| *ALT* | IU/L | 28 | **45** |  | 28 | **32** |  | 30 | **43** |  | 33 | **110** |  | 32 | **43** |
| *AST* | IU/L | 82 | **125** |  | 72 | **118** |  | 75 | **183** |  | 80 | **200** |  | 83 | **85** |
| *GGT* | IU/L | <3 | **<3** |  | <3 | **<3** |  | <3 | **<3** |  | <3 | **<3** |  | <3 | **<3** |
| *Creatine Kinase* | IU/L | 1417 | **544** |  | 904 | **526** |  | 1047 | **951** |  | 1140 | **498** |  | 1306 | **275** |
| *Sodium* | mmol/L | 147 | **145** |  | 147 | **148** |  | 148 | **152** |  | 147 | **148** |  | 149 | **153** |
| *Potassium* | mmol/L | 7.3 | **>10.0** |  | 6.5 | **<10.0** |  | 7.1 | **9.5** |  | 6.9 | **>10.0** |  | 7.1 | **9.9** |
| *Chloride* | mmol/L | 104 | **106** |  | 102 | **105** |  | 103 | **108** |  | 103 | **108** |  | 103 | **110** |
| *Bicarbonate* | mmol/L | 19 | **29** |  | 19 | **29** |  | 18 | **27** |  | 19 | **28** |  | 17 | **29** |
| *Amylase* | IU/L | 3174 | **3413** |  | 3023 | **5632** |  | 3074 | **3448** |  | 2865 | **3569** |  | 2852 | **2979** |
| *Lipase* | IU/L | 48 | **53** |  | 45 | **819** |  | 48 | **46** |  | 52 | **53** |  | 47 | **50** |
| *Icteric Index* |  | 0 | **0** |  | 0 | **0** |  | 0 | **0** |  | 0 | **0** |  | 0 | **0** |
| *Hemolysis* |  | 35 | **67** |  | 28 | **42** |  | 40 | **56** |  | 43 | **106** |  | 28 | **56** |
| *Lipemia Index* |  | 13 | **19** |  | 14 | **10** |  | 12 | **10** |  | 12 | **13** |  | 13 | **13** |

**Supplementary Table 3: Coagulation values**

|  | *International Units (IU)* | *Saline* | *Vehicle* | *Paclitaxel* | *TRAP-Paclitaxel* |
| --- | --- | --- | --- | --- | --- |
| *Prothrombin Time* | Sec | 11.7 | 11.3 | 11.3 | 11.1 |
| *Activated Partial Thromboplastin TIME* | Sec | 23.8 | 22.6 | 24.6 | 21.7 |
| *DDimer* | ng/mL | <135 | <135 | <135 | <135 |
| *Platelets* | 10^3/µL | 981 | 937 | 511 | 1546 |
| *Fibrinogen* | mg/dL | 222 | 213 | 317 | 255 |

**Supplementary Table 4: Urinalysis values**

|  |  | *SALINE* | |  | | | *VEHICLE* | |  | *PACLITAXEL* | |  | *TRAP* | |  | *TRAP-PACLITAXEL* | |  |
| --- | --- | --- | --- | --- | --- | --- | --- | --- | --- | --- | --- | --- | --- | --- | --- | --- | --- | --- |
| TEST | International Units (IU) | Day 0 | Day 28 |  | | | Day 0 | Day 28 |  | Day 0 | Day 28 |  | Day 0 | Day 28 |  | Day 0 | Day 28 |  |
| *pH* |  | 7 | **8** |  | | 6.5 | | **6.5** |  | 6.5 | **6.5** |  | 6 | **7** |  | 6.5 | **7** |  |
| *protein dipstick* | mg/dL | 30 A | **500 A** |  | | 30 A | | **30 A** |  | 30 A | **100 A** |  | 30 A | **30 A** |  | 30 A | **30 A** |  |
| *Glucose* | mg/dL | Normal | **Normal** |  | | Normal | | **Normal** |  | Normal | **Normal** |  | Normal | **Normal** |  | Normal | **Normal** |  |
| *Ketones* | mg/dL | 15 A | **5 A** |  | | 15 A | | **15 A** |  | 15 A | **15 A** |  | 15 A | **15 A** |  | 15 A | **15 A** |  |
| *Bilirubin Dipstick* |  | Negative | **Negative** |  | | Negative | | **Negative** |  | Negative | **Negative** |  | Negative | **Negative** |  | Negative | **Negative** |  |
| *Blood* | /µL | Negative | **250A** |  | | 10 A | | **10 A** |  | 10 A | **10 A** |  | 50 A | **Negative** |  | 10 A | **Negative** |  |
| *Urine Color* |  | Yellow | **Light Yellow** |  | | Yellow | | **Yellow** |  | Yellow | **Gold A** |  | Yellow | **Yellow** |  | Yellow | **Yellow** |  |
| *Urine Clarity* |  | Clear | **Slightly Cloudy A** | | Clear | | | **Clear** |  | Clear | **Slightly Cloudy A** | | Clear | **Clear** |  | Clear | **Clear** |  |
